# Obesity Selectively Increases Intraoperative Risk in Left-Sided Colon Cancer Surgery: A Retrospective Cohort Study

**DOI:** 10.1007/s00384-025-04989-5

**Published:** 2025-08-25

**Authors:** Simon Xu, Rathin Gosavi, Yat Cheung Chung, William Teoh, T. C. Nguyen, Geraldine Ooi, Vignesh Narasimhan

**Affiliations:** 1 Department of Colorectal Surgery, Monash Health, David Street, Dandenong, Melbourne, VIC 3193 Australia; 2 Department of Colorectal Surgery, Cabrini Hospital, Melbourne, Australia; 3 Department of Surgery (School of Clinical Sciences at Monash Health), Monash University, Melbourne, Australia

**Keywords:** Obesity, Colon cancer, Surgical outcomes, BMI, Postoperative complications

## Abstract

**Background:**

Obesity is traditionally viewed as a risk factor for adverse surgical outcomes. This study evaluated whether obesity (BMI ≥ 30 kg/m^2^) independently affected intraoperative and postoperative outcomes following colon cancer resection, and whether these effects varied by anatomical site.

**Methods:**

A retrospective cohort study was conducted of consecutive patients who underwent colon cancer resection at a single institution from 2015 to 2022. Patients were stratified by BMI (≥ 30 kg/m^2^ vs < 30 kg/m^2^) and further grouped by urgency (elective or emergency) and anatomical subsite (right- vs left-sided). Primary outcomes were intraoperative complications, severe postoperative morbidity (Clavien–Dindo ≥ III), conversion to open surgery, ICU admission, and 30-day mortality. Multivariate logistic regression was used to adjust for confounders.

**Results:**

Among the 737 patients, 33.5% were obese (BMI ≥ 30). Obese patients were younger and had higher rates of hypertension (55% vs 46%, p = 0.01), diabetes (25% vs 16%, p < 0.01), and respiratory disease (22% vs 11%, p < 0.01). In the overall cohort, obesity was not associated with increased rates of intraoperative complications, severe postoperative complications, conversion to open surgery, or 30-day mortality. In elective resections, obesity was independently associated with increased likelihood of ICU admission (aOR 1.82, 95% CI 1.08–3.09; p = 0.02), while in emergent resections obesity was independently associated with higher intra-operative complications (aOR 2.18, 95% CI 1.19–3.97; p = 0.01). Stratified analysis by resection site revealed that obesity was an independent risk factor associated with intraoperative complications (aOR 1.89, 95% CI 1.03–3.47; p = 0.04) and ICU admission (aOR 3.17, 95% CI 1.61–6.23; p < 0.01) following left-sided colectomy, but not right-sided surgery.

**Conclusions:**

Obesity was not associated with adverse outcomes following colon cancer surgery overall. However, when stratified by anatomical subsite, obesity was independently associated with increased perioperative risk in left-sided resections. These findings support a more nuanced approach to operative planning and perioperative risk stratification.

## Introduction

Obesity affects more than 650 million adults globally and remains a rising public health concern [1]. In Australia, over 30% of adults are classified as obese (body mass index (BMI) ≥ 30 kg/m^2^), with implications for surgical safety and outcomes [2]. In colorectal cancer surgery, obesity is widely perceived to increase operative complexity, lengthen procedural time, and heighten peri-operative risk [3–5]. However, evidence remains inconsistent.

Several studies have reported increased blood loss, surgical site infections, and conversion to open surgery in obese patients [6, 7], while others have shown no significant differences or even lower mortality and complication rates, a phenomenon sometimes described as the “obesity paradox” [8]. These discrepancies may reflect methodological limitations, including inconsistent adjustment for comorbidities, operative urgency, and approach.

Anatomical and technical differences between colon and rectal surgery further complicate generalisations. In rectal cancer, excess mesorectal fat and a narrow bony pelvis substantially limit exposure and increase operative risk in obese patients [9]. This was supported by recent data from our institution showing that obesity independently predicted intraoperative complications and higher rates of conversion to open surgery in rectal resections [10]. In contrast, colonic resections are typically performed in more accessible operative fields, and the specific impact of obesity in this context remains uncertain.

This study aimed to determine whether obesity, defined as BMI ≥ 30 kg/m^2^, independently predicts adverse intraoperative and postoperative outcomes in patients undergoing colon cancer resection. While prior studies have often considered colon resections as a homogeneous group, technical complexity varies substantially depending on tumour location. Left-sided procedures, such as left hemicolectomies or high anterior resections frequently require splenic flexure mobilisation, some pelvic dissection and anastomosis formation in deeper operative fields. These challenges may be amplified in obese patients due to excess visceral adiposity.

We hypothesised that obesity would not uniformly impact perioperative outcomes across all colon cancer resections. By leveraging a large, prospectively maintained institutional database and stratifying by resection site and surgical urgency, we sought to determine whether the effect of BMI on operative outcomes differs between right- and left-sided colon cancer surgery.

## Methods

### Study design

This was a retrospective cohort study conducted at Monash Health, a tertiary academic institution in Melbourne, Australia. The study included consecutive patients who underwent colon cancer resection between 1 January 2015 and 31 December 2022. Data were extracted from a prospectively maintained colorectal cancer surgery database and cross-referenced with electronic medical records to ensure completeness and accuracy.

### Eligibility Criteria

Patients aged 18 years or older who underwent surgical resection for histologically confirmed colonic adenocarcinoma were eligible for inclusion. Eligible procedures included right hemicolectomy, transverse colectomy, left hemicolectomy, high anterior resection, Hartmann’s procedure, and total or subtotal colectomy. Patients undergoing rectal cancer resection, non-curative surgery, or surgery for metastatic disease were excluded.

### Patient Stratification

Patients were stratified into two cohorts based on body mass index (BMI): BMI < 30 kg/m^2^ and BMI ≥ 30 kg/m^2^. Baseline demographic variables, comorbidities (hypertension, diabetes, ischaemic heart disease, and respiratory disease), American Society of Anesthesiologists (ASA) physical status, surgical urgency (elective versus emergency), and operative approach (laparoscopic or open) were recorded. Robotic surgery was not performed at our institution during the study period; all minimally invasive cases were laparoscopic. BMI was calculated from documented preoperative height and weight measurements recorded in the anaesthesia pre-assessment clinic. Resection site was categorised as either right sided (right/transverse colectomy) or left-sided resection (including left hemicolectomy, high anterior resection, and Hartmann’s procedure), based on procedural documentation.

### Variables and Definitions

Baseline variables included age, sex, ASA physical status classification, smoking status, and comorbidities (hypertension, diabetes mellitus, ischaemic heart disease, respiratory disease). Operative details included surgical urgency (elective or emergency), operative approach (laparoscopic, open, or conversion to open), and procedure type.

### Outcome measures

The primary outcome was the incidence and severity of intra-operative adverse events, classified using the CLASSIntra system. Secondary outcomes included conversion to open surgery, postoperative complications (graded by the Clavien-Dindo classification within 30 days), intensive care unit (ICU) admission, and hospital length of stay (LOS). Operative duration was not consistently available and was therefore not included as a measure of technical complexity.

### Intraoperative complications

Intraoperative adverse events were graded using the validated ClassIntra classification system [11], a standardised framework for assessing adverse events occurring during surgery and anaesthesia. This system classifies intraoperative events from Grade 0 (ideal operative course) to Grade V (intraoperative death), based on severity and the intervention required. Grades I and II reflect minor to moderate deviations manageable without lasting consequences, whereas Grades III–IV involve more serious events requiring significant intraoperative intervention or posing immediate risk to life. Two independent reviewers (SX and RG) assigned ClassIntra grades based on operative and anaesthetic records. Discrepancies were resolved by consensus with senior author oversight. Conversion to open surgery, although not classified within ClassIntra, was documented as a distinct outcome.

### Post operative complications

Postoperative complications were classified according to the Clavien–Dindo system [12], with Grade III or higher considered severe. Other secondary outcomes included ICU admission, LOS, and 30-day mortality.

### Statistical Analysis

Categorical variables were compared using Pearson’s chi-squared or Fisher’s exact tests, as appropriate. Continuous variables were analysed using Student’s t-tests or Mann–Whitney U tests based on distributional characteristics. Logistic regression was used to evaluate the association between obesity and outcomes, with multivariate models adjusted for age, sex, ASA classification, smoking status, operative urgency, surgical approach, and comorbidities.

Subgroup analyses were performed to evaluate whether the association between obesity and outcomes varied by clinical context. Specifically, we stratified patients by surgical urgency (elective vs emergency) and by anatomical resection site (right or transverse colectomy vs left hemicolectomy, high anterior resection, or Hartmann’s procedure).

Multivariate logistic regression models were constructed for each subgroup using the same covariates as the primary analysis. The interaction between BMI and resection site was explored to assess effect modification. A two-sided p-value of less than 0.05 was considered statistically significant. All analyses were performed using Stata version 18 (StataCorp, College Station, TX, USA).

### Ethics

This study was approved by the Monash Health Human Research Ethics Committee (Reference: RES-23–0000-816Q). The need for informed consent was waived due to the retrospective nature of the study and use of de-identified data.

## Results

### Patient characteristics

A total of 737 patients underwent colon cancer resection during the study period, comprising 490 (66%) with BMI < 30 kg/m^2^ and 247 (34%) with BMI ≥ 30 kg/m^2^. Patients in the obese group were significantly younger (mean age 64.4 vs 69.7 years, p < 0.01) and less likely to be male (60% vs 46%, p = 0.01). Obese patients had higher rates of hypertension (55% vs 46%, p = 0.01), diabetes (25% vs 16%, p < 0.01), ischaemic heart disease (28% vs 19%, p < 0.01), and respiratory disease (22% vs 11%, p < 0.01). ASA class III or IV was more prevalent in the obese cohort (70% vs 60%, p < 0.01). Baseline characteristics are summarised in Table 1.

**Table 1:** Baseline characteristics by body mass index (BMI)

Variables	Total (n = 737)	BMI ≥ 30 (n = 490)	BMI < 30 (n = 247)	p-value
Age, years (mean ± sd)	67.0 ± 13.9	69.7 ± 12.5	64.4 ± 13.2	< 0.01
Sex (male, %)	389 (53)	150 (60)	114 (46)	0.01
Comorbidities (n for yes, %)				
Smoking	285 (39)	191 (39)	94 (38)	0.90
Hypertension	359 (49)	223 (46)	136 (55)	0.01
Ischaemic heart disease	159 (22)	91 (19)	68 (28)	< 0.01
Diabetes	139 (19)	77 (16)	62 (25)	< 0.01
Respiratory disease	111 (15)	56 (11)	55 (22)	< 0.01
Use of antiplatelet therapy	83 (11)	60 (12)	23 (9)	0.03
Surgical approach (number, %)				0.64
Laparoscopic	462 (63)	304 (62)	158 (64)	
Open	167 (23)	116 (24)	51 (21)	
Conversion from lap to open	108 (15)	70 (14)	38 (15)	
Operative urgency (n, %)				0.11
Elective	460 (62)	296 (60)	164 (66)	
Emergency	277 (38)	194 (40)	83 (34)	
ASA (n, %)				< 0.01
1	28 (4)	26 (5)	2 (1)	
2	243 (33)	170 (35)	73 (29)	
3	407 (55)	259 (53)	148 (60)	
4	59 (8)	35 (7)	24 (10)	
Operation type (n, %)				0.61
Right/transverse hemicolectomy	375 (51)	247 (51)	128 (52)	
High anterior resection/Left hemicolectomy/Hartmanns	286 (39)	192 (39)	94 (38)	
Total/subtotal colectomy	51 (10)	51 (10)	25 (10)	

### Overall Perioperative Outcomes

There were no significant differences in intra-operative complications between obese and non-obese patients (29% vs 27%, p = 0.50; adjusted OR 1.24, 95% CI 0.85–1.81). Rates of conversion to open surgery were identical in both groups (19%, p = 0.85; adjusted OR 1.06, 95% CI 0.65–1.72).

Postoperative complications occurred in 8% of all patients (Grade III or higher), with no significant difference between obese and non-obese cohorts (6% vs 9%, p = 0.11; adjusted OR 0.72, 95% CI 0.37–1.43).

There was no difference in ICU admissions between cohorts (8% vs 5%, p = 0.07; adjusted OR 1.41, 95% CI 0.94–2.12). Mean length of ICU stay (2.1 vs 2.9 days, p = 0.42) and overall hospital stay (9.3 vs 10.4 days, p = 0.31) did not differ significantly. Thirty-day mortality was 1% in both groups (p = 0.59). These results are detailed in Table 2.

**Table 2:** Primary and secondary outcome by BMI

Variables	Total (n = 737)	Body mass index (BMI) in 2 categories (kg/m2)					
		**BMI < 30 (n = 490)**	**BMI ≥ 30 (n = 247)**	**OR unadjusted (95%CI)**	**p-value (unadjusted)**	OR adjusted* (95%CI)	p-value (adjusted*)
BMI (median, IQR)	**27.5 (8.1)**						
Any intra-op Complications (yes, %)	202 (27)	130 (27)	72 (29)	1.13 (0.81–1.60)	0.50	1.24 (0.85–1.81)	0.27
Grade 0	535 (73)	360 (73)	175 (71)				
Grade 1	29 (4)	16 (3)	13 (5)				
Grade 2	138 (19)	92 (19)	46 (19)				
Grade 3	28 (4)	19 (4)	9 (4)				
Grade 4	7 (1)	3 (1)	4 (2)				
No post-op complications or CD Grade I/II	679 (92)	446 (91)	233 (94)				
CD Grade III	37 (5)	27 (6)	10 (4)				
CD Grade IV	13 (2)	11 (2)	2 (1)				
CD Grade V	8 (1)	6 (1)	2 (1)				
CD Grade >/= III	58 (8)	44 (9)	14 (6)	0.61 (0.33–1.13)	0.11	0.72 (0.37–1.43)	0.35
Conversion rate (yes, %)	108 (19)	70 (19)	38 (19)	1.04 (0.67 ± 1.62)	0.85	1.06 (0.65–1.72)	0.81
ICU admission (yes, %)	209 (28)	130 (27)	79 (32)	1.30 (0.92–1.80)	0.12	1.35 (0.91–2.07)	0.15
Length of ICU admission in days (mean ± sd)	2.6 ± 2.9	2.9 ± 3.4	2.1 ± 1.6		0.42		0.29
30-days mortality (yes,%)	8 (1)	6 (1)	2 (1)		0.63		0.59
Length of hospital day in days (mean ± sd)	10.0 ± 8.4	10.4 ± 8.7	9.3 ± 7.9		0.12		0.31

^*^Adjusted to age, sex, ASA, smoking, operation type, surgical approach (except for conversion rate), operation urgency

### Elective Surgery

Among the 460 patients who underwent elective resection, intra-operative complication rates were similar between obese and non-obese groups (26% vs 28%, adjusted OR 0.89, 95% CI 0.56–1.42, p = 0.62).

Postoperative complications were infrequent in this subgroup (5%), with no difference based on BMI (4% vs 6%, adjusted OR 0.48, 95% CI 0.17–1.37, p = 0.17).

ICU admission was significantly more common among obese patients (29% vs 21%, p = 0.04), and this association persisted after adjustment (adjusted OR 1.82, 95% CI 1.08–3.09, p = 0.02).

Length of stay and 30-day mortality remained comparable between groups (Table 3).

**Table 3:** Subgroup analysis – Elective surgery only

Variables	Total (n = 460)	Body mass index (BMI) in 2 categories (kg/m2)					
		**BMI < 30 (n = 296)**	**BMI ≥ 30 (n = 164)**	**OR unadjusted (95%CI)**	**p-value (unadjusted)**	OR adjusted* (95%CI)	p-value (adjusted*)
BMI (median, IQR)	**27.9 (8.3)**						
Any intra-op Complications (yes, %)	126 (27)	84 (28)	42 (26)	0.87 (0.56–1.34)	0.53	0.89 (0.56–1.42)	0.62
Any post-op complications** (yes, %)	23 (5)	17 (6)	6 (4)	0.62 (0.24–1.61)	0.33	0.48 (0.17–1.37)	0.17
Conversion rate (yes, %)	69 (17)	41 (15)	28 (19)	1.32 (0.78–2.25)	0.30	1.47 (0.82–2.62)	0.20
Laparoscopic, including conversion (yes, %)	416 (90)	270 (91)	146 (89)				
Open (yes,%)	44 (10)	26 (9)	18 (11)				
ICU admission (yes, %)	109 (24)	61 (21)	48 (29)	1.59 (1.03–2.47)	0.04	1.82 (1.08–3.09)	0.02
Length of ICU admission in days (mean ± sd)	2.1 ± 1.8	2.3 ± 1.8	1.9 ± 1.7		0.50		0.97
30-days mortality (yes,%)	5 (1)	4 (1)	1 (1)		0.49		0.73
Length of hospital day in days (mean ± sd)	8.1 ± 5.5	8.2 ± 5.3	8.0 ± 5.9		0.71		0.23

^*^Adjusted to age, sex, ASA, smoking, operation type, surgical approach (except for conversion rate)

^**^post-op complication includes Clavien-Dindo grade III or above

### Emergency Surgery

In emergency cases (n = 277), obese patients had a significantly higher rate of intra-operative complications (36% vs 24%, p = 0.04), which remained significant after adjustment (adjusted OR 2.18, 95% CI 1.19–3.97, p = 0.01).

There was no significant difference in conversion to open surgery, rates of postoperative complications, ICU admission, and mortality did not differ between groups (Table 4).

**Table 4:** Subgroup analysis – Emergency surgery only

Variables	Total (n = 277)	Body mass index (BMI) in 2 categories (kg/m2)					
		**BMI < 30 (n = 99)**	**BMI ≥ 30 (n = 178)**	**OR unadjusted (95%CI)**	**p-value (unadjusted)**	OR adjusted* (95%CI)	p-value (adjusted*)
BMI (median, IQR)	**26.7 (7.7)**						
Any intra-op Complications (yes, %)	76 (27)	46 (24)	30 (36)	1.82 (1.04–3.18)	0.04	2.18 (1.19–3.97)	0.01
Any post-op complications^**^(yes, %)	35 (13)	27 (14)	8 (10)	0.66 (0.28–1.52)	0.33	0.66 (0.27–1.61)	0.36
Conversion rate (yes, %)	39 (25)	29 (28)	10 (20)	0.65 (0.29–1.46)	0.29	0.57 (0.24–1.34)	0.20
Laparoscopic, including conversion (yes, %)	154 (56)	104 (54)	50 (60)				
Open (yes,%)	123 (44)	90 (46)	33 (40)				
ICU admission (yes, %)	100 (36)	69 (36)	31 (37)	1.08 (0.63–1.84)	0.78	1.25 (0.66–2.35)	0.49
Length of ICU admission in days (mean ± sd)	3.2 ± 3.7	3.5 ± 4.3	2.5 ± 1.4		0.30		0.24
30-days mortality (yes,%)	3 (1)	2 (1)	1 (1)		0.78		0.89
Length of hospital day in days (mean ± sd)	13.2 ± 11.1	13.7 ± 11.4	12.0 ± 10.3		0.26		0.45

^*^Adjusted to age, sex, ASA, smoking, operation type, surgical approach (except for conversion rate)

^**^post-op complication includes Clavien-Dindo grade III or above

### Analysis by Resection Type

To assess whether anatomical location modified the effect of obesity, outcomes were stratified by tumour location. Among patients who underwent right sided resections (n = 375), obesity was not independently associated with intra-operative complications (adjusted OR 0.83, 95% CI 0.48–1.43, p = 0.50), ICU admission (adjusted OR 1.12, 95% CI 0.64–1.98, p = 0.69), or severe postoperative morbidity (Supplementary Table).

In contrast, in patients undergoing left-sided resections (n = 286), obesity was independently associated with significantly increased risk of both intra-operative complications (adjusted OR 1.89, 95% CI 1.03–3.47, p = 0.04) and ICU admission (adjusted OR 3.17, 95% CI 1.61–6.23, p < 0.01) (Table 5).

**Table 5:** Subgroup Analysis (Left Hemicolectomy/Anterior Resection/Hartmanns)

Variables	Total (n = 94)	Body mass index (BMI) in 2 categories (kg/m2)					
		**BMI < 30 (n = 192)**	**BMI ≥ 30**	**OR unadjusted (95%CI)**	**p-value (unadjusted)**	OR adjusted* (95%CI)	p-value (adjusted*)
BMI (median, IQR)	**27.2 (7.6)**						
Any intra-op Complications (yes, %)	80 (28)	49 (26)	31 (33)	1.44 (0.84–2.36)	0.19	1.89 (1.03–3.47)	0.04
Any post-op complications (yes, %)	23 (8)	16 (8)	7 (7)	0.89 (0.35–2.23)	0.80	0.93 (0.34–2.57)	0.89
Conversion rate (yes, %)	48 (23)	29 (21)	19 (28)	1.48 (0.76–2.90)	0.25	1.74 (0.84–3.59)	0.14
Laparoscopic, including conversion (yes, %)	208 (73)	140 (73)	68 (72)				
Open (yes,%)	78 (27)	26 (28)	52 (27)				
ICU admission (yes, %)	85 (30)	48 (25)	37 (39)	1.95 (1.15–3.30)	0.01	3.17 (1.61–6.23)	< 0.01
Length of ICU admission in days (mean ± sd)	2.3 ± 2.0	2.3 ± 2.2	2.3 ± 1.7		0.83		0.62
30-days mortality (yes,%)	1	1	0				
Length of hospital day in days (mean ± sd)	10.1 ± 7.9	10.0 ± 7.4	10.2 ± 8.8		0.10		0.07

^*^Adjusted to age, sex, ASA, smoking, operation urgency, surgical approach (except for conversion rate)

^**^post-op complication includes Clavien-Dindo grade III or above

No significant differences in postoperative complication rates or 30-day mortality were observed between BMI groups within either resection category.

## Discussion

In this retrospective cohort of 737 colon cancer resections, obesity was not associated with increased intraoperative complications, severe postoperative morbidity, or mortality overall. Despite higher rates of cardiometabolic comorbidities, outcomes in obese patients were comparable to non-obese peers. However, when stratified by urgency and anatomical site, obesity independently predicted increased perioperative risk, particularly in emergency and left-sided resections.

These observations partially align with prior institutional data in rectal cancer, where obesity was associated with higher intraoperative risk, conversion rates, and ICU admission [10]. In contrast, obesity-related risk in colon cancer was more anatomically restricted, with no difference in conversion rates or postoperative complications. This likely reflects differing technical challenges; rectal surgery is constrained by a narrow pelvis and mesorectal fat, whereas colon surgery offers broader exposure, reducing the impact of visceral adiposity.

One of the most clinically meaningful findings in this study is that anatomical context appears to modulate the effect of BMI on risk. Left-sided colectomies, which involve splenic flexure mobilisation and deeper pelvic dissection, may be disproportionately affected by excess mesenteric fat. These demands likely underlie the increased intraoperative complications and ICU utilisation observed in obese patients. Right-sided resections, with greater access and less pelvic dissection, were not similarly impacted.

ICU admission following elective surgery was more common in obese patients, likely reflecting proactive care decisions rather than true complications. By contrast, emergency surgery in obese patients was associated with higher intraoperative complication rates, potentially reflecting limited physiological reserve, unoptimised comorbidities, and increased technical difficulty. These findings support a more nuanced approach to preoperative planning. Rather than using BMI alone to stratify risk or guide operative approach, anatomy-specific planning and optimisation strategies, prehabilitation, metabolic support, and structured perioperative respiratory planning may improve outcomes.

While obesity was associated with increased intraoperative complications in both left-sided and emergency resections, this did not translate into severe postoperative morbidity. Several explanations may account for this observation. First, many intraoperative events were managed definitively at the time of surgery, without sustained physiological consequences. Second, high levels of intraoperative vigilance, senior surgeon involvement, and access to intensive postoperative monitoring may have mitigated downstream clinical impact. This pattern suggests that intraoperative complications in this context were more reflective of technical difficulty than of systemic decompensation. The dissociation between intraoperative and postoperative outcomes reinforces the value of real-time recognition and intraoperative problem-solving in maintaining postoperative safety, even in technically challenging cases.

While BMI remains a widely used metric in surgical research and risk stratification, its limitations as a surrogate for body composition are increasingly recognised. It fails to account for visceral fat distribution, sarcopenia, or systemic inflammation [13, 14]. This is particularly relevant given our finding that obesity conferred site-specific risk in left-sided but not right-sided resections. A recent meta-analysis of over 300,000 patients across 57 studies demonstrated that visceral adiposity and reduced skeletal muscle mass are more accurate predictors of perioperative complications and long-term oncological outcomes than BMI alone [3]. Prior studies have similarly shown that these indices are more closely associated with postoperative morbidity and survival [7, 15]. While not yet routinely used, tools such as CT body composition analysis or bioimpedance may improve preoperative risk modelling and individualised planning [16].

This study has several limitations. The retrospective design introduces risks of selection bias and unmeasured confounding. Although multivariable models adjusted for key covariates, we were unable to control for nutritional status, frailty, or surgeon experience. In addition, cancer staging data was not routinely available and therefore not included in the analysis. BMI was calculated from preoperative weight and height but not verified with imaging-based body composition metrics. Operative duration, a relevant surrogate for technical complexity, was not recorded uniformly and therefore not included in the analysis. While prolonged operative time is frequently observed in patients with obesity [17], its absence did not appear to influence short-term postoperative outcomes in this cohort. Nonetheless, it remains a potential contributor to intraoperative difficulty, particularly in left-sided resections. Additional intraoperative factors such as use of extra ports, need for assistant retractors, or variation in surgical approach were not captured, and may have introduced procedural nuance not reflected in the dataset. Furthermore, subgroup analyses, particularly in the emergency and left-sided cohorts, may be underpowered to detect small differences in postoperative outcomes, raising the possibility of type II error. In addition, robotic-assisted colectomies are increasingly adopted in some health systems and may mitigate the technical challenges encountered in obese patients undergoing left-sided resections. However, access remains limited in many Australian and New Zealand public hospitals. Finally, this was a single-centre study performed in a high-volume academic setting with widespread laparoscopic access, which may limit the external validity of these findings.

Future work should explore whether targeted interventions such as GLP-1 receptor agonists, short-term weight loss, or prehabilitation can reduce risk in obese patients, and whether body composition metrics can be embedded into standard preoperative workflows.

## Conclusion

Obesity was independently associated with increased intraoperative complications in patients undergoing left-sided and emergency colon cancer resections. This association was not observed in right-sided surgery or in elective cases overall. Future studies should incorporate imaging-based body composition metrics to improve risk stratification and inform anatomy-specific operative strategies.

### ASA: the American Society of Anesthesiologist physical status classification system

OR: Odds ratio, IQR: Inter-quartile range, CD: Clavien-Dindo classification, ICU: Intensive care unit.

OR: Odds ratio, IQR: Inter-quartile range, CD: Clavien-Dindo classification, ICU: Intensive care unit.

OR: Odds ratio, IQR: Inter-quartile range, CD: Clavien-Dindo classification, ICU: Intensive care unit.

OR: Odds ratio, IQR: Inter-quartile range, CD: Clavien-Dindo classification, ICU: Intensive care unit.

## Data Availability

No datasets were generated or analysed during the current study.
